# Physical exercise augmented cognitive behaviour therapy for older adults with generalised anxiety disorder (PEXACOG): a feasibility study for a randomized controlled trial

**DOI:** 10.1186/s13030-023-00280-7

**Published:** 2023-07-19

**Authors:** Kristine Sirevåg, S. H. Stavestrand, T. Sjøbø, T. B. Endal, H. M. Nordahl, E. Andersson, I. H. Nordhus, Å. Rekdal, K. Specht, Å. Hammar, A. Halmøy, J. Mohlman, H. Hjelmervik, J. F. Thayer, A. Hovland

**Affiliations:** 1 Solli DPS, Osvegen 15 Nesttun, 5228 Bergen, Norway; 2 Department of Clinical Psychology, University of Bergen, P.O. Box 7800, Bergen, NO-5020 Norway; 3 Department of Mental Health, Norwegian University of Science and Technology, Trondheim, NO-7030 Norway; 4 The Swedish School of Sport and Health Sciences, GIH, Stockholm, 5626 SE-114 86 box Sweden; 5 Department of Neuroscience, Karolinska Institutet, Stockholm, 171 177 Sweden; 6 Faculty of Medicine, University of Oslo, P.O. Box 1078, Blindern, Oslo, NO-0316 Norway; 7 Faculty of humanities, social sciences and education, UiT The Arctic University of Norway, Tromsø, Norway; 8 Department of Biological and Medical Psychology, University of Bergen, P.O. Box 7807, Bergen, NO- 5020 Norway; 9 Division of Psychiatry, Haukeland University Hospital, Bergen, Norway; 10 Department of Psychiatry, Haukeland University Hospital, Kronstad DPS, P.O. Box 1400, Bergen, NO- 5021 Norway; 11 Department of Clinical Medicine, University of Bergen, P.O. Box 7804, Bergen, NO-5020 Norway; 12 Department of Psychology, William Paterson University, 300 Pomton Road, Wayne, NJ 07470 USA; 13 School of Health Sciences, Kristiania University College, Kalfarveien 78c, Bergen, 5022 Norway; 14 Department of Psychological Science, The University of California, Irvine, 4201 Social and Behavioral Sciences Gateway, Irvine, CA 92697 USA

**Keywords:** Generalised anxiety disorder, GAD, Cognitive behaviour therapy, CBT, Physical exercise, Older adults, Feasibility study

## Abstract

**Background:**

Generalised anxiety disorder (GAD) is a frequent and severe disorder among older adults. For older adults with GAD the effect of the recommended treatment, cognitive behaviour therapy (CBT), is reduced. Physical exercise (PE) may enhance the effect of CBT by improving cognitive function and increasing levels of brain-derived neurotrophic factor (BDNF), a predictor of the effect of CBT in patients with anxiety. The aim of the study was to assess the feasibility of a randomized controlled trial (RCT) investigating treatment effect of the combination of CBT and PE for GAD in a sample of older adults, including procedures for assessment and treatment.

**Methods:**

Four participants aged 62–70 years (*M* = 65.5, *SD* = 3.2) with a primary diagnosis of GAD were included. Participants received 15 weeks of PE in combination with 10 weeks of CBT. Participants completed self-report measures, and clinical, biological, physiological and neuropsychological tests at pre-, interim- and post-treatment.

**Results:**

Procedures, protocols, and results are presented. One participant dropped out during treatment. For the three participants completing, the total adherence to PE and CBT was 80% and 100%, respectively. An independent assessor concluded that the completers no longer fulfilled the criteria for GAD after treatment. Changes in self-report measures suggest symptom reduction related to anxiety and worry. The sample is considered representative for the target population.

**Conclusions:**

The results indicate that combining CBT and PE for older adults with GAD is feasible, and that the procedures and tests are suitable and manageable for the current sample.

**Trial registration:**

ClinicalTrials.gov, NCT02690441. Registered on 24 February 2016, https://clinicaltrials.gov/ct2/show/NCT02690441.

**Supplementary Information:**

The online version contains supplementary material available at 10.1186/s13030-023-00280-7.

## Introduction

Generalised anxiety disorder (GAD) is a severe disorder characterised by profound and uncontrollable worry and associated with symptoms such as restlessness, fatigue, muscle tension, irritability, difficulty concentrating, and sleep problems [1].

Cognitive behaviour therapy (CBT) is the recommended treatment [2] and has been found to be more effective as treatment for GAD compared to passive control conditions [3]. However older adults are less responsive to treatment than younger adults [4, 5]. The reduced effect of CBT for older adults with GAD may be explained by age-related cognitive changes or decline in executive functions [6–8]. An approach to enhance the effect of CBT has been to add physical exercise (PE) to the treatment [9]. PE improves cognitive function [10], increases brain volume in prefrontal and temporal cortices in non-demented older adults [11] and is associated with increased brain plasticity and cognitive function through the mechanisms of the neurotrophin brain-derived neurotropic factor (BDNF; 12). BDNF has a protective effect on brain health [13], and levels are reduced in patients with depression and anxiety [14, 15]. An increase in BDNF levels through PE can enhance the ability to learn and remember new material. These effects are hypothesised to an enhancement of the effect of CBT in older adults with GAD [16].

There are no previous studies investigating the combination of CBT and PE in older adults with GAD. Previous studies have shown that PE is feasible in the treatment of anxiety disorders [17–19] and also specifically in GAD [20–22]. The main aim of this study is to investigate the feasibility of the combination of CBT and PE in a sample of older adults with GAD as a precursor to a RCT. The RCT study protocol has been published elsewhere [16].

### Objectives

The objectives of the study are (1) to evaluate the feasibility of combining CBT and PE for older adults with GAD, (2) to assess the feasibility of test protocols and scheduled time frames and (3) to troubleshoot screening and inclusion procedures. Results from the questionnaires and physical manipulation checks are discussed.

## Methods

### Design

**Fig. 1:**
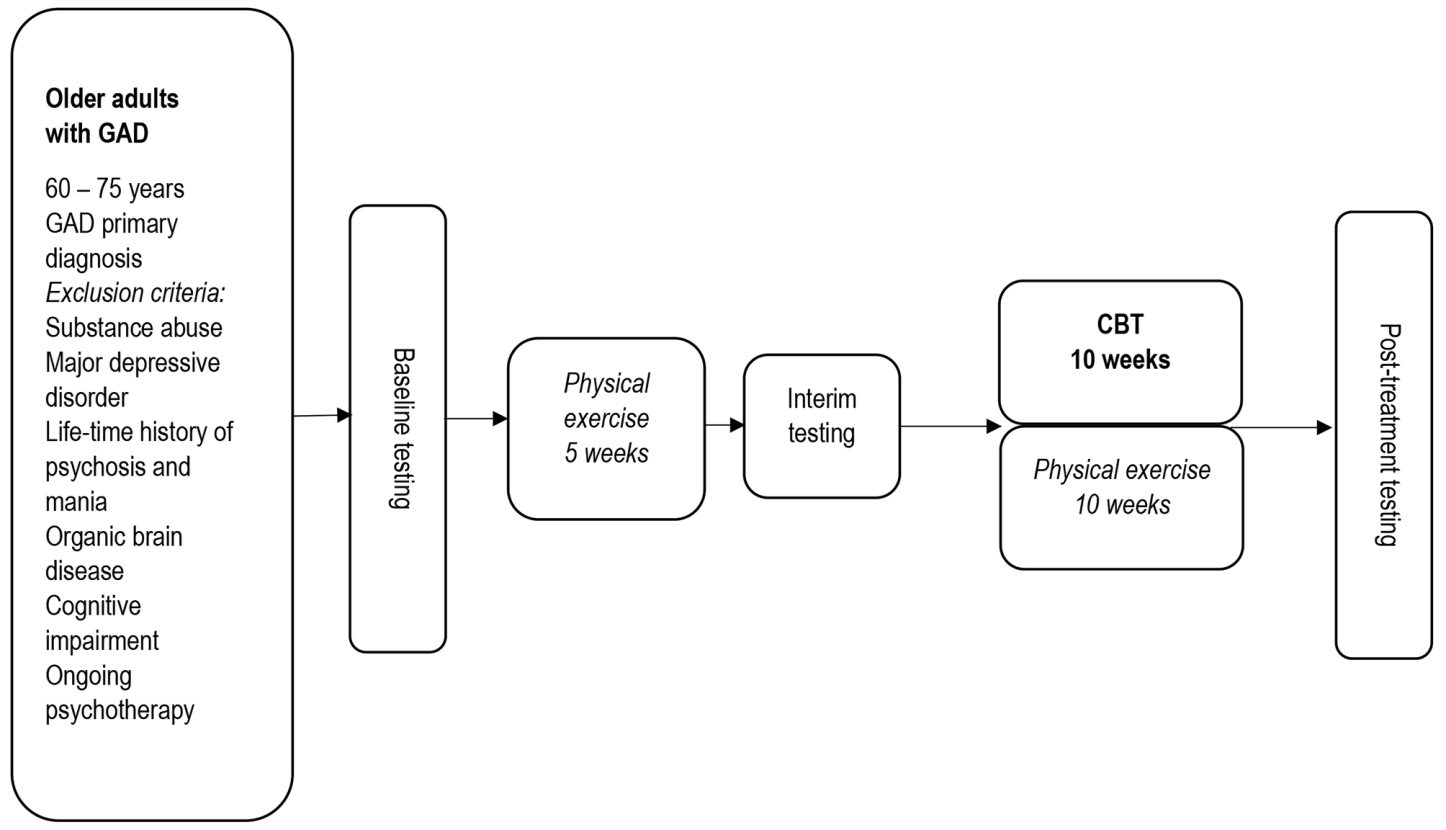
Feasibility study design Note. * DNA sampled only at pre-treatment testing. ** Actiwatch, MRI and neuropsychological tests not administered at Interim testing

The study had a single-arm design and all participants received CBT combined with PE. The participants completed pre-treatment, interim and post-treatment measures. Figure 1 displays the feasibility study design.

### Participants

The participants were recruited from an outpatient, psychiatric secondary care-clinic. Inclusion criteria were (1) age between 60 and 75 years; (2) a primary diagnosis of GAD as evaluated by the Anxiety Disorders Interview Schedule for DSM-IV (ADIS-IV; 23). Exclusion criteria were: (1) substance abuse; (2) habitual use of benzodiazepines; (3) changes in the dose of other psychotropic medication during the study; (4) medical conditions that preclude participation in physical testing and/or PE; (5) severe major depression as determined by the Mini-International Neuropsychiatric Interview (M.I.N.I.; 24, 25), a structured interview for psychiatric disorders in the DSM-IV; (6) life-time history of psychosis and/or mania; (7) participation in other ongoing psychotherapy; (8) organic brain disease; (9) a score of 25 or less on the Mini Mental State Examination (MMS-E; 26), a measure for grading cognitive state and (10) physical exercise of moderate intensity of 60 min or more of two or more sessions per week during an average week for the last three months.

### Assessment for eligibility

For eligibility assessment potential participants met twice with a project coordinator (a clinical psychologist) for completion of the Generalized Anxiety Disorder Questionnaire (GAD-7) [27], the GAD-module in the ADIS-IV interview [23], the M.I.N.I [24, 25]., and the MMS-E [26]. Level of physical activity was assessed by registering physical activity during an average week for the last three months.

### Interventions

**Cognitive behaviour therapy**. The CBT intervention consisted of an initial session focused on providing rapport and information, and 10 weekly sessions of 60 min of individual manualised CBT. The CBT intervention was based on Borkovec’s protocol for treatment of GAD [28]. Prior to the feasibility study, the therapists had completed one practice treatment with the CBT manual which were video rated by an independent assessor using the Cognitive Therapy Adherence and Competence Scale [29].

**Physical exercise.** The PE intervention consisted of 15 weekly supervised and unsupervised individual sessions of resistance and aerobic training, each scheduled to last 45–60 min. Both resistance and aerobic training intensity levels progressed every five weeks. Supervised sessions were led by physiotherapists or an occupational therapist.

### Assessments

**Questionnaires.** The following inventories was administered: The Penn State Worry Questionnaire (PSWQ) [30], the Beck Anxiety Inventory (BAI) [31], the Beck Depression Inventory – II (BDI-II) [32], the Generalized Anxiety Disorder Questionnaire (GAD-7) [27], the Geriatric Anxiety Inventory (GAI) [33], the Bergen Insomnia Scale (BIS) [34], the Credibility/Expectancy Questionnaire [35], the International Physical Activity Questionnaire (IPAQ-short) [36, 37] and a questionnaire for treatment satisfaction post treatment.

**Physical measures.** The Ekblom-Bak submaximal cycle ergometer test [38] and the Five-Minute Pyramid test [39] were used for assessing aerobic fitness. Four endurance muscle strength tests measured functional physical strength; the Biering-Sørensen test [40] for back muscle strength, a Timed sit-to-stand test (50 times) as a measure of lower-extremity strength, a 45-degree sit-up test for core muscle strength and a dumbbell arm press test for arm and shoulder strength.

**Other measures.** All measures for the RCT were tested during the feasibility study. The results from these measures are not indicative of the feasibility, and thus not reported. A description of all measures is available elsewhere [16].

### Test procedures

**Fig. 2:**
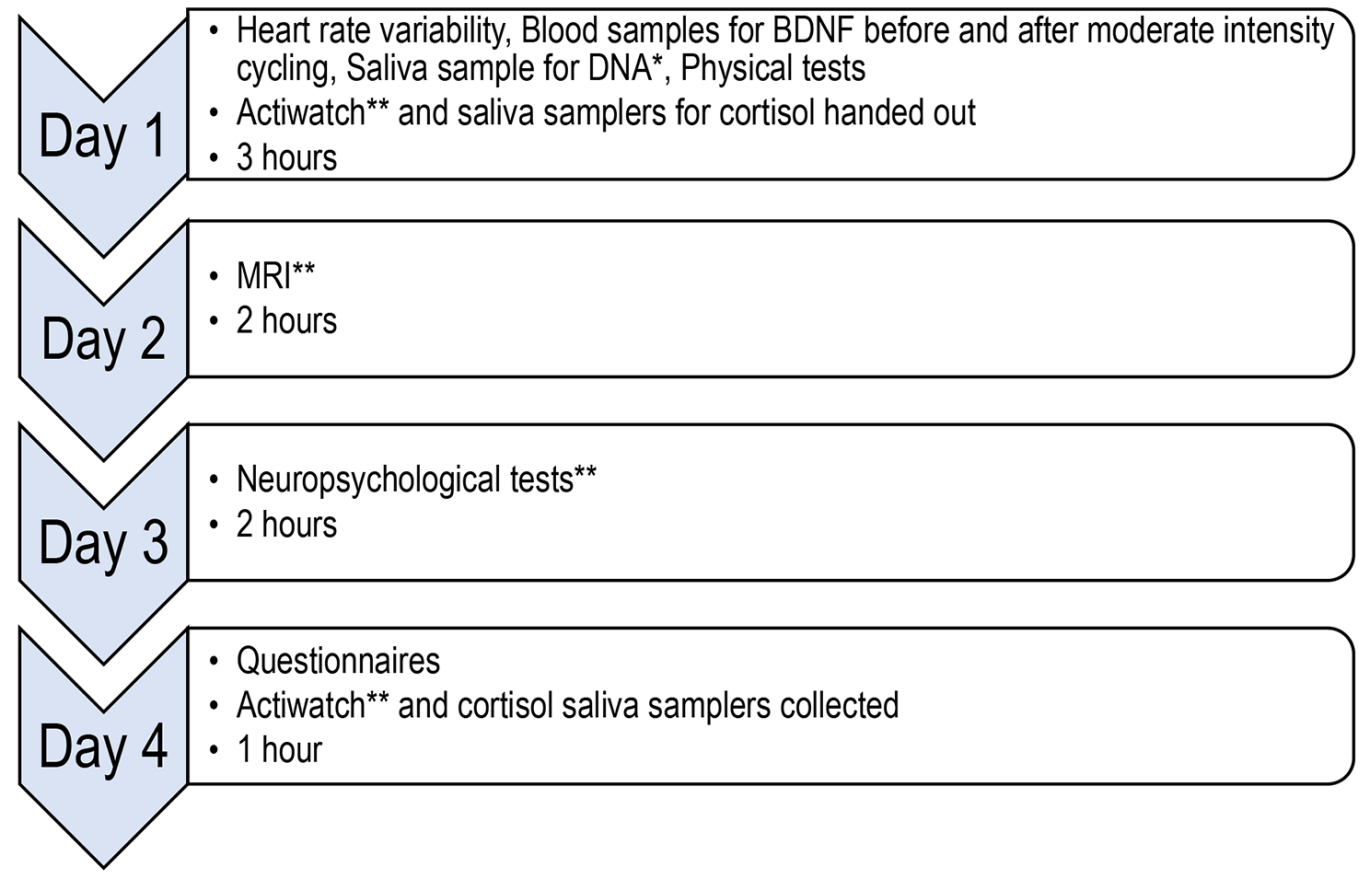
Distribution of measures

Pre- and post-measures were distributed across four days (Fig. 2 displays the distribution of measures). Pre-measures were conducted within two weeks of recruitment and post-measures were conducted within two weeks and completed not more than two weeks after finishing treatment. The interim test was distributed over two days. The interim test was conducted within one week, and not more than one week after finishing pre-treatment.

### Statistics

Means (*M*) and standard deviations (*SD*) for questionnaires and test results were calculated with IBM SPSS Statistics, version 26 [41] to determine the reliable change index (RCI). The RCI was calculated using the procedure described by Evans and colleagues [42] and Zahra and Hedge [43]. Criteria C from Evans and colleagues [42], which estimates greater likelihood of the patient being in the normative distribution than a clinical distribution after treatment was used to determine the clinical cut off point for the varying measures. The mean and standard deviations for the norm and clinical populations were calculated using the source papers for the Penn State Worry Questionnaire [44], the Beck Anxiety Inventory [45], the Beck Depression Inventory II [46, 47], the Geriatric Anxiety Inventory [33], the Generalized Anxiety Disorder Questionnaire [48], and the Bergen Insomnia Scale [49]. Likewise, the reliability of each measure was the Cronbach’s alpha for each measure. Due to the small sample size in the current study, alpha from the abovementioned source papers were used.

## Results

### Participant flow

**Fig. 3:**
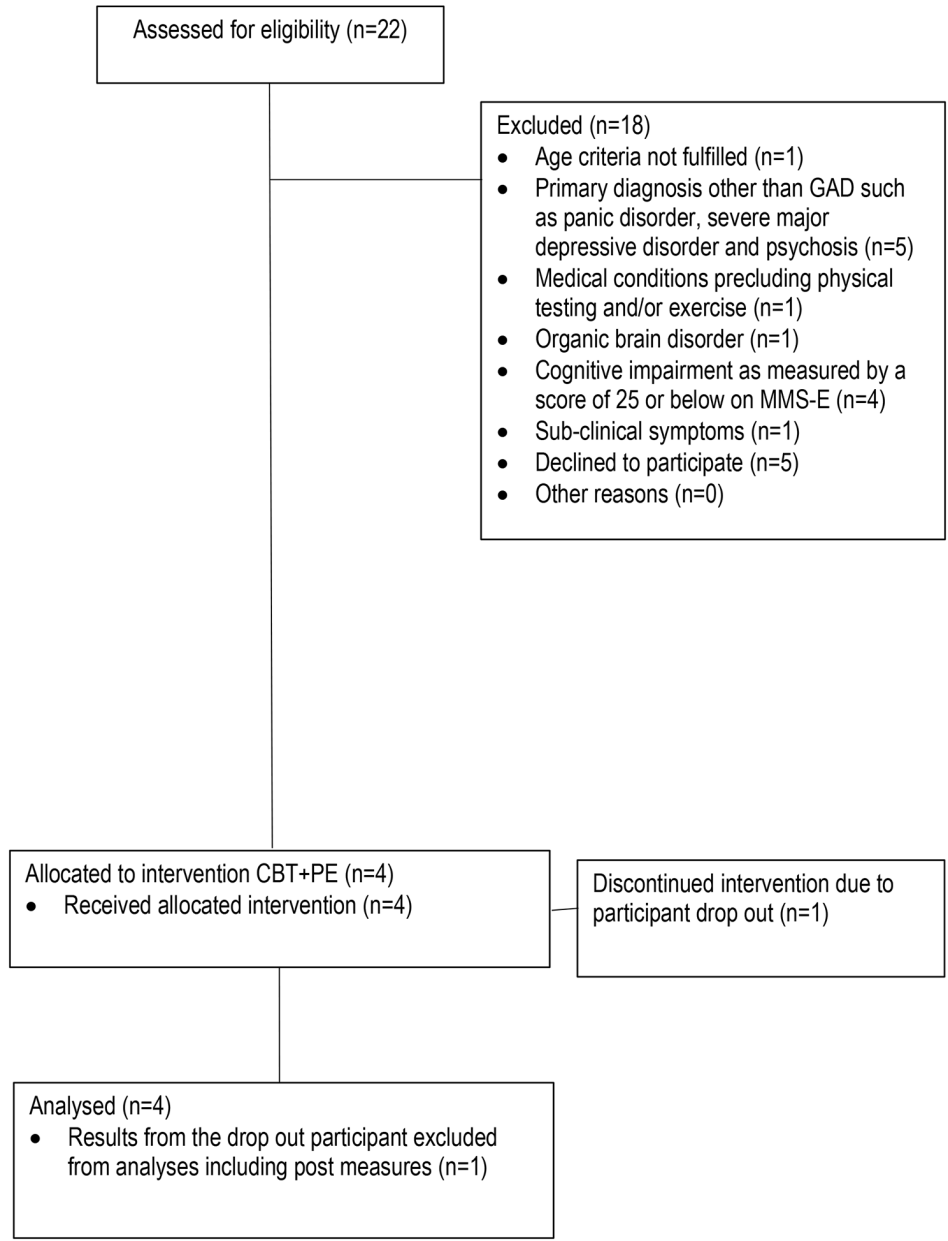
Feasibility CONSORT Flow Diagram

A total of 22 potential participants were assessed for participation (see Fig. 3 for study flow), and nine were considered eligible according to the inclusion- and exclusion criteria. Five subjects decline to participate, with reference to the expected demand of the overall treatment protocol. Four participants were included in the study. The remaining 18 potential participants were excluded according to the exclusion criteria. Excluded patients needing other treatment and/or care were ensured this.

One participant dropped out seven weeks after reporting challenges with combining ongoing treatment with household chores. Results from the three participants who completed the treatment are included in analyses of changes due to intervention. Results from the drop-out participant are included in reports of baseline characteristics. Baseline data displaying demographic and clinical characteristics of the sample is seen in Table 1.

**Table 1:** Sample characteristics

Parameter	*N* = 4
Sex	
Male	50%
Female	50%
Age	
Mean	65.5
SD	3.2
Range	8
Marital status	
Partner	75%
Single	25%
Level of education	
Elementary school – max. 10 years	25%
High school – max. 13 years	50%
Certificate of apprenticeship	25%
Tapering of benzodiazepines	75%
Activity level at screening	
Everyday activity level	*M* = 61–89 min
Physical exercise	*M* = up to 29 min
GAD-7* at screening	*M* = 16.5
MMS-E** score	*M* = 29.75
M.I.N.I*** comorbid diagnosis	
Major Depressive Disorder – ongoing or previous	100%
Suicidality	75%
Panic disorder – with or without agoraphobia	75%

*GAD-7: Generalized Anxiety Disorder Questionnaire – 7 [31]

**MMS-E: Mini Mental Status Examination [30]

***M.I.N.I: Mini International Neuropsychiatric Interview [28, 29]

### Feasibility data

Completing participants attended 10 CBT sessions (100%), while the drop-out participant attended three CBT sessions (30%). Completers attended 93.3% and 73.3% of supervised and unsupervised PE sessions, respectively, resulting in a total adherence to PE of 80% for completers. The drop-out participant completed seven supervised PE sessions and 12 unsupervised sessions and attended all treatment while still participating. Individual rates of adherence are displayed in Table 2.

**Table 2:** Adherence to treatment

Participant	Completed CBT-sessions (%)^a^	Avg. weekly CBT-sessions	PE – supervised (%)^b^	PE – unsupervised (%)^c^	Total PE sessions (%)^d^	Avg. weekly PE sessions
1*	3 (30)	0.3	7 (46.7)	12 (40)	19 (42.2)	1.3
2	10 (100)	1	15 (100)	15 (50)	30 (66.7)	2.0
3	10 (100)	1	12 (80)	24 (80)	36 (80)	2.4
4	10 (100)	1	15 (100)	27 (90)	42 (93.3)	2.8
Tot. avg. completers	10 (100)	1	14 (93.3)	22 (73.3)	36 (80)	2.4
Tot. avg. with dropout	8.3 (82.5)	0.83	12.3 (81.7)	19.5 (65)	31.8 (70.6)	2.1

* Drop-out

^a^ Proportion of the 10 scheduled weekly CBT-sessions. Percentages in parenthesis

^b^ Proportion of the 15 scheduled weekly supervised PE-sessions. Percentages in parenthesis

^c^ Proportion of the 30 scheduled weekly unsupervised PE-sessions. Percentages in parenthesis

^d^ Proportion of the 45 scheduled weekly supervised and unsupervised PE-sessions. Percentages in parenthesis

Completers adhered 100% to test-protocols at pre-, interim and post-measurement points. The drop-out participant completed all pre- and interim tests and hence had a total of 67% adherence to test-protocols.

All pre- and post-tests were finished within two weeks, respectively. All interim tests were completed within one week, and a maximum of one week after completion of the pre-treatment period. All post-tests started a maximum of two weeks after completed treatment.

### Treatment outcomes

Results from each participant are presented in Tables 3 and 4.

**Table 3:** Outcome measures

Questionnaire	Pre-treatment	Post-treatment	Change pre-post	RCI
**PSWQ**				
1	74	n/a	n/a	n/a
2	56	47	-9	-2.27*
3	73	57	-16	-4.04*
4	70	56	-14	-3.53*
*Clinical cut-off: 41.2*				
**BAI**				
1	26	n/a	n/a	n/a
2	12	6^b^	-6	-2.29*
3	25	10^b^	-15	-5.72*
4	18	3^b^	-15	-5.27*
*Clinical cut-off: 11.9*				
**BDI-II**				
1	24	n/a	n/a	n/a
2	10^a^	2^b^	-8	-1.07
3	22	15	-7	-0.94
4	44	13^b^	-31	-4.16*
*Clinical cut-off: 14.9*				
**GAI**				
1	20	n/a	n/a	n/a
2	15	7	-8	-4.25*
3	19	17	-2	-1.06
4	19	11	-8	-4.25*
*Clinical cut-off: 5.5*				
**GAD-7**				
1	13	n/a	n/a	n/a
2	9^a^	4^b^	-5	-2.4*
3	17	10	-7	-3.4*
4	14	3^b^	-11	-5.3*
*Clinical cut-off: 9.7*				
**BIS**				
1	21	n/a	n/a	n/a
2	0^a^	6^b^	6	0.9
3	18	25	7	1.03
4	24	9^b^	-15	-2.2*
*Clinical cut-off: 16.5*				

*Note. Clinical cut-off* refers to the estimated transition point from healthy to clinical state and vice versa

a Pre-score below clinical cut-off

b Post-score below clinical cut-off

*RCI values greater than 1.96 in either direction denote statistically significant change at the *p < .05*

**Table 4:** Results on the physical manipulation checks

Physical test	Pre-treatment	Interim	Post-treatment	Change pre-post (%)
5-minute pyramid test^a^				
1 (woman)	25.4		n/a	n/a
2 (man)	21.7		24.1	11.06
3 (man)	27.9		30.9	10.75
4 (woman)	22.1		24.4	10.41
Ekblom-Bak cycle test^b^				
1	29.4	31.8	n/a	n/a
2	23.3	28.0	29.3	25.75
3	26.6	28.6	31.2	17.30
4	22.7	24.2	25.3	11.5
Biering-Sørensen test^c^				
1	128		n/a	n/a
2	80		75	-6.25
3	65		81	24.62
4	119		146	22.69
Timed sit-to-stand test^d^				
1	0.52	0.72	n/a	n/a
2	0.67	0.67	0.74	10.45
3	0.63	0.66	0.75	19.05
4	0.65	0.68	0.86	32.31
45° sit-up test^e^				
1	137	148	n/a	n/a
2	38	40	68	78.95
3	30	65	75	150.00
4	44	63	105	138.64
Dumbbell arm press test^f^				
(3/5 kg – women/men)				
1	71		n/a	n/a
2	44		40	-9.09
3	50		75	50
4	50		100	100

^a^ Values are VO2_max_ (ml/kg/min) pre and post treatment

^b^ Values are VO2_max_ (ml/kg/min) pre and post treatment

^c^ Values are seconds held in a static position

^d^ Values are speed (number of repetitions per second)

^e^ Values are seconds held in a static position

^f^ Values are number of repetitions

**Clinical rating.** None of the completing participants were assessed as fulfilling criteria for GAD after completion of the treatment after an independent assessment with the GAD-module in ADIS-IV [23].

**Primary outcome measure.** Table 3 displays individual scores on the PSWQ [34]. Completers had a statistically reliable change as shown by the RCI on the PSWQ from pre- to post-measures. None had a clinically significant change according to the clinical cut-off point as determined by Method C from Evans and colleagues [42].

**Secondary outcome measures – questionnaires.** Individual scores on the outcome measures BAI [31], BDI-II [32], GAI [33], GAD-7 [27], and BIS [34] are displayed in Table 3. The table also shows the RCI and the clinical cut-off point as determined by Method C from Evans and colleagues [42]. Completers in general showed a statistically reliable change in scores on the anxiety measures BAI, GAI and GAD-7. They additionally changed from above to below clinical cut-off on the BAI and GAD-7. The results for the BDI-II and BIS are mixed, with two out of three participants not showing a statistically reliable change nor a reduction in scores that changed from the clinical to the normal range. All participants had a reduction in scores on the BDI-II. On BIS, two participants had elevated scores from pre- to post-measures.

**Participant treatment satisfaction and expectancy/credibility to treatment.** Completers filled out a treatment satisfaction questionnaire. Participants reported satisfaction with treatment, that they had changed “quite much” in a positive direction, and that other people had noticed this change. Participants reported that they were “mostly satisfied” with the changes. One participant reported “exclusively positive effects” and one reported “no unfortunate effects of any matter”. One reported “some unfortunate effects” and referred to a strain injury that incurred during PE. Participants reported that the treatment fitted their problems well. All three participants reported that the treatment was “somewhat demanding”, and that they “complied overall well” with treatment demands.

Participants had a mean pre-treatment score of 7.8 (*SD* = 0.59) on the Expectancy/Credibility scale [35] and an increase in mean post-treatment score to 8.2 (*SD* = 1.46).

### Manipulation check for physical exercise

Results from physical testing pre- and post-treatment showed that completers improved their aerobic physical fitness with an average of 10.7% on the Five-Minute Pyramid test [39] and 18.2% on the The Ekblom-Bak submaximal cycle ergometer test [38]. The completing participants improved their physical strength with an average of 13.7% on the Biering-Sørensen test, 20.6% on the Timed sit-to-stand test, 122.5% on the 45-degree sit-up test, and 47% on the Dumbbell arm press test. One participant had a decrease in results on the Biering-Sørensen test and Dumbbell arm test as a consequence of engaging in heavy physical activity the day before testing. Individual test results are shown in Table 4.

## Discussion

### Adherence to treatment, test protocols and time frames

The current study showed that the CBT + PE protocol for older adults with GAD is feasible. It appears somewhat more challenging for the participants to adhere to unsupervised PE sessions than the supervised PE sessions. Evaluation indicated that the drop-out was not related to structure or organization of the combined treatment. Additionally, completers reported adequate treatment satisfaction, that the treatment was somewhat demanding, but that they complied quite well with the treatment.

Test protocols were evaluated as feasible. The high adherence may be explained by the participants` motivation to learn about their own physical and mental health, and positive attitudes towards research.

### Feasibility of screening and inclusion

The study resulted in two two-hour screening appointments, contrary to an initial three hours per appointment. Regarding the inclusion process, some of the potential participants with symptoms of GAD were excluded as they had a primary panic disorder or severe major depressive disorder. Included participants had symptoms of comorbid panic disorder (75%) and depression (100%) but GAD was assessed as the primary disorder. The comorbidity between GAD and other anxiety disorders and depression is known [50]. There has been critique towards studies aiming at including participants with “pure” GAD, with the argument that the generalizability of the results of these studies are limited as comorbidity is frequent [51]. In the current study, besides primary panic disorder and severe major depression, comorbid anxiety disorders and depression were not reasons for exclusion.

Evaluation of the inclusion process led to the addition of antipsychotic medication to the exclusion criteria due to potential sedative effects that can prevent the treatment effect of CBT. Tapering benzodiazepines was successful through the established procedures.

As a result of the drop out, inclusion procedures were aimed towards not including participants in testing and/or treatment in the time leading up to holidays or events.

### Effects of treatment

Scores on the PSWQ [30] showed a statistically, but not clinically, reliable change, according to the cut-off as determined by the analysis in this study. This implies that the scores have not changed from a clinical to a non-clinical population. Also, diagnostic assessment after treatment showed that none of the participants had GAD.

Johnco, Wuthrich, Brenes, Wetherell and Mohlman [52] discuss whether the PSWQ is a suboptimal measure for evaluating treatment outcome in geriatric GAD, as there does not exist a clear cut-off or benchmark for the PSWQ to determine patients in remission and patients that show response to treatment. The authors found that a score of 51 or below is optimal for defining remission status on the PSWQ, and a 9% reduction or > 4-point reduction is optimal for assessing treatment response on the PSWQ. With these criteria, one of the participants in the current study would have been characterised as in remission, and two of the participants would be characterised as having a treatment response as they had 20% reduction on the scores on the PSWQ from pre to post treatment.

Participants showed the greatest reduction in scores on anxiety measures, and least reduction in scores of depression and sleep problems. As the CBT protocol is targeted at reducing anxiety, this is not a surprising finding. The change in depression scores can be associated with the rumination component of depression, which resembles worry – the core component of GAD. A treatment that taps into worry might also influence rumination. In addition, PE has been found to have a robust effect on depressive symptoms [53]. As participants in the current study engaged in PE, this may explain the change in depressive symptoms. However, as this study did not include a control group, the change can also be due to non-treatment related factors.

The manipulation checks overall yielded expected results after 15 weeks of PE and indicate that the intensity of the exercise intervention is sufficient to further investigate the effect on treatment outcomes.

### Limitations

The procedures for recruitment for the RCT and the CBT + placebo control condition as described in the RCT design [16] were not tested. Furthermore, the small sample limits the generalisability of results of feasibility. In assessing the feasibility of the study design, one limitation is that only four out of 22 assessed subjects were included in the study. Due to our recruitment strategy, the participants that we assessed were mainly in treatment at the local psychiatric facility. As such, they had other treatment options. We have not investigated this further, but we believe these factors to be relevant in understanding the low inclusion percentage in the feasibility study. To preserve the data quality needed to answer research questions in the RCT, we chose to keep the exclusion criteria from the feasibility, although this strategy can be a limitation to the ecological validity of the study.

### Clinical implications

- The current protocol of CBT + PE, as well as test protocols, for older adults with GAD is feasible.
- The study indicates treatment effect of the combined treatment for older adults with GAD.

### Electronic supplementary material

Below is the link to the electronic supplementary material.

Supplementary Material 1

## Declarations

The Regional Committee for Medical and Health Research Ethics, region North in Norway has approved the study, reference number 2015/2189, approved 11. November 2015. All important protocol modifications are reported to the Regional Committee for Medical and Health Research Ethics for approval. Participants were given written and oral information about the study before enrolment. All participants have signed informed consent before enrolment in the study.

Datasets are available from the corresponding author on reasonable request.
