# Correction to: Physical exercise augmented cognitive behaviour therapy for older adults with generalised anxiety disorder (PEXACOG): a feasibility study for a randomized controlled trial

**DOI:** 10.1186/s13030-023-00284-3

**Published:** 2023-08-04

**Authors:** Kristine Sirevåg, S. H. Stavestrand, T. Sjøbø, T. B. Endal, H. M. Nordahl, E. Andersson, I. H. Nordhus, Å Rekdal, K. Specht, Å Hammar, A. Halmøy, J. Mohlman, H. Hjelmervik, J. F. Thayer, A. Hovland

**Affiliations:** 1 Solli DPS, Osvegen 15 Nesttun, 5228 Bergen, Norway; 2 Department of Clinical Psychology, University of Bergen, P.O. Box 7800, Bergen, NO-5020 Norway; 3 Department of Mental Health, Norwegian University of Science and Technology, Trondheim, NO-7030 Norway; 4 The Swedish School of Sport and Health Sciences, GIH, Stockholm, 5626 SE-114 86 box Sweden; 5 Department of Neuroscience, Karolinska Institutet, Stockholm, 171 177 Sweden; 6 Faculty of Medicine, University of Oslo, P.O. Box 1078, Blindern, Oslo, NO-0316 Norway; 7 Faculty of humanities, social sciences and education, UiT The Arctic University of Norway, Tromsø, Norway; 8 Department of Biological and Medical Psychology, University of Bergen, P.O. Box 7807, Bergen, NO- 5020 Norway; 9 Division of Psychiatry, Haukeland University Hospital, Bergen, Norway; 10 Department of Psychiatry, Haukeland University Hospital, Kronstad DPS, P.O. Box 1400, Bergen, NO- 5021 Norway; 11 Department of Clinical Medicine, University of Bergen, P.O. Box 7804, Bergen, NO-5020 Norway; 12 Department of Psychology, William Paterson University, 300 Pomton Road, Wayne, NJ 07470 USA; 13 School of Health Sciences, Kristiania University College, Kalfarveien 78c, Bergen, 5022 Norway; 14 Department of Psychological Science, The University of California, Irvine, 4201 Social and Behavioral Sciences Gateway, Irvine, CA 92697 USA

**Correction: BioPsychoSocial Med 17, 25 (2023)**

10.1186/s13030-023-00280-7

Following publication of the original article [1], the authors reported errors in the author names, which have been corrected from:

I. H. K. Nordhus to I. H. Nordhus, Rekdal to Å. Rekdal, and Hammar to Å. Hammar.

The original article [1] has been updated.
