# Supplementary material for: Physical exercise augmented cognitive behaviour therapy for older adults with generalised anxiety disorder (PEXACOG): a feasibility study for a randomized controlled trial
Source: Biopsychosoc Med. 2023 Jul 19;17:25. doi: 10.1186/s13030-023-00280-7 (PMC10357630; doi:10.1186/s13030-023-00280-7)
Supplement: Supplementary file 1 — Supplementary Material 1 [file 13030_2023_280_MOESM1_ESM.docx]

**Manuscript BPSM-D-22-00052
Response to Reviewers**

Dear Editors-in-Chief and Reviewers,

Thank you for giving us the opportunity to submit a revised draft of the manuscript “Physical exercise augmented cognitive behaviour therapy for older adults with generalised anxiety disorder (PEXACOG): A feasibility study for a randomized controlled trial”. We appreciate you and the Reviewers for providing valuable comments, leading to improvements of the manuscript. The authors have carefully considered the comments and have put an effort in addressing each of them. We have incorporated the suggestions made by the reviewers. Those changes are highlighted within the manuscript. Please see below, in blue, for a point-by-point response to the reviewers’ comments and concerns. All page numbers refer to the revised manuscript file with highlighted changes.

**Reviewers’ Comments to the Authors:**

**Reviewer #1**

This is a feasibility study for a randomized controlled trial on the combination of physical exercise + cognitive behavior therapy for older adults with generalized anxiety disorders.

I have no comments.

**Author response:** Thank you for reviewing the manuscript.

**Reviewer #2**

The authors report a feasibility study on cognitive behavioral therapy plus physical exercise for older patients with generalized anxiety disorder. The manuscript is straightforward and well-organized. The concept is novel and would contribute to the existing literature. A few minor points should be modified.

**1.** Only 14% (3/22) of potential participants met the eligible criteria and ultimately completed the study. This suggests that applying this intervention to real-world clinical practices seems difficult, and the effectiveness is not promising in the current form. The authors should acknowledge this limitation or discuss the potential expansion of the target of intervention.

**Author response:** Thank you for this valuable suggestion. The section detailing “Participant flow” on p. 7 has been updated with the following:

“A total of 22 potential participants were assessed for participation (see Figure 3 for study flow), and nine were considered eligible according to the inclusion- and exclusion criteria. Five subjects decline to participate, with reference to the expected demand of the overall treatment protocol.”

A consideration of the external validity and potential implications for the recruitment to the overall RCT has been added to p. 12 of the manuscript with the following wording:

“In assessing the feasibility of the study design, one limitation is that only four out of 22 assessed subjects were included in the study. Due to our recruitment strategy, the participants that we assessed were mainly in treatment at the local psychiatric facility. As such, they had other treatment options. We have not investigated this further, but we believe these factors to be relevant in understanding the low inclusion percentage in the feasibility study. To preserve the data quality needed to answer research questions in the RCT, we chose to keep the exclusion criteria from the feasibility, although this strategy can be a limitation to the ecological validity of the study.”

**2.** Criterion C requires mean and SD from individuals with disease status and those with healthy status (Evans et al., 1998). The authors should clearly note how to get mean and SD in both groups in the Statistical Analysis section. Likewise, the authors should also describe how to get the reliability oof each measurement to quantify the RCIs.

**Author response:** Thank you for noticing this. We have clarified this in the statistical analysis section of the manuscript. Please refer to the paragraph below, or p. 6-7 in the manuscript.

“The mean and standard deviations for the norm and clinical populations were calculated using the source papers for the Penn State Worry Questionnaire (Beck et al., 1995), the Beck Anxiety Inventory (Morin et al., 1999), the Beck Depression Inventory II (Segal et al., 2008; Steer et al., 1999), the Geriatric Anxiety Inventory (Pachana et al., 2007), the Generalized Anxiety Disorder Questionnaire (Spitzer et al., 2006), and the Bergen Insomnia Scale (Pallesen et al., 2008). Likewise, the reliability of each measure was the Cronbach’s alpha for each measure. Due to the small sample size in the current study, alpha from the abovementioned source papers were used.”
